# Denosumab in multiple myeloma patients with severe renal impairment: a multicenter phase 2 study

**DOI:** 10.1038/s41408-026-01656-y

**Published:** 2026-11-02

**Authors:** Elizabeth K. O’Donnell, Omar Nadeem, Jacob P. Laubach, Giada Bianchi, Andrew R. Branagan, Brea C. Lipe, Paul G. Richardson, Ehsan Malek, Robb S. Friedman, Ajay K. Nooka, Robert Redd, Noopur S. Raje, Andrew J. Yee

**Affiliations:** 1 Mass General Brigham Cancer Institute, Boston, MA USA; 2 Harvard Medical School, Boston, MA USA; 3 Present Address: Dana-Farber Cancer Institute, Boston, MA USA; 4 University of Rochester Medical Center, Rochester, NY USA; 5 University Hospitals of Cleveland, Cleveland, OH USA; 6 Present Address: Roswell Park Comprehensive Cancer Center, Buffalo, NY USA; 7 Newton-Wellesley Hospital, Newton, MA USA; 8 Present Address: Dana-Farber Brigham Cancer Center at South Shore Health, South Weymouth, MA USA; 9 Winship Cancer Institute, Emory University, Atlanta, GA USA

**Keywords:** Myeloma, Phase II trials

To the editor:

Lytic bone lesions are a defining characteristic of multiple myeloma (MM) and one of its most significant clinical consequences. Bone-modifying agents, including the bisphosphonates zoledronic acid and pamidronate, as well as the receptor activator of nuclear factor-κB ligand (RANKL) monoclonal antibody denosumab, are a mainstay of bone supportive care [1, 2]. However, a major limitation of zoledronic acid and pamidronate is nephrotoxicity, with guidelines recommending caution in patients with creatinine clearance <30 mL/min [3]. This is particularly relevant in MM where renal dysfunction is common at presentation and at relapse [4]. In contrast, denosumab has a significantly lower risk of kidney injury compared with zoledronic acid in a large, randomized study in MM that led to its approval [5]. However, patients with creatinine clearance <30 mL/min were excluded from this trial, limiting application of these findings to this subgroup. Given the unmet need for bone supportive care in MM patients with renal impairment, we conducted a prospective, multicenter, phase 2 trial of denosumab in this patient population (NCT02833610).

Eligible participants were adults (age ≥18 years) with newly diagnosed or relapsed MM with a creatinine clearance of <30 mL/min and were not eligible for a bisphosphonate. Renal function was assessed using either the Chronic Kidney Disease Epidemiology Collaboration (CKD-EPI) equation or the Cockcroft-Gault equation, per local institutional practice. Prior denosumab, or treatment with an intravenous bisphosphonate in the past three months, were exclusion criteria. Patients received open-label denosumab 120 mg subcutaneously every four weeks for a total of 12 cycles. All patients received calcium supplementation (e.g., calcium citrate 2000 mg daily) and vitamin D 1000 IU daily. Prior to starting treatment, a 25-OH vitamin D level ≥30 ng/mL was required. The primary endpoint was change in serum C-terminal telopeptide of type I collagen (CTX) levels at 3 months, a biochemical marker of bone resorption. Secondary endpoints included safety and adverse events.

Between April 2017 and March 2022, we enrolled 24 patients across six sites, and 22 patients received denosumab and were analyzed for outcomes. Two of the 24 patients did not receive denosumab due to rapidly progressive disease or withdrawal of consent. The study closed early due to slow accrual. The baseline characteristics are summarized in Table 1. Median age was 68 years (47–82), and 12 (55%) had newly diagnosed MM. The median creatinine clearance was 23.8 mL/min (range 10-28.6), and 9 (41%) were on dialysis.

**Table 1:** Baseline characteristics.

	*N* = 22
Age, median (range)	68 (47–82)
Male/Female	13 (59%)/9 (41%)
Disease status	
Newly diagnosed	12 (55%)
Relapsed/refractory	10 (45%)
Race	
Black or African-American	1 (5%)
White	21 (95%)
Ethnicity	
Hispanic	1 (5%)
Non-Hispanic	21 (95%)
Performance status	
0	4 (18%)
1	15 (68%)
2	3 (14%)
Hemodialysis	9 (41%)
Creatinine (mg/dL), median (range)	3.1 (2.0–7.7)
Creatinine clearance (mL/min), median (range)	23.8 (10–28.6)
Calcium (mg/dL), median (range)	9.2 (8.3–10.3)
25-OH vitamin D (ng/mL), median (range)	48.5 (30–-89)

Two patients did not start denosumab and are not included above.

Patients received a median of 11 doses of denosumab (range 1–12). The median follow-up was 2.8 years. Thirteen patients completed denosumab treatment. Of the 9 patients who did not complete the study, 3 withdrew consent, 2 had progressive disease, 1 moved out of the region, 1 developed ONJ, 1 patient had a pending dental extraction, and 1 was at the treating physician’s discretion.

The median serum CTX at baseline was 864 pg/mL (range 465–4009) and at 3-month follow-up, it was reduced to 292 pg/mL (range 86-3037), representing a significant median 73% reduction from baseline (*n* = 18, Wilcoxon signed-rank test, *p* = 0.0002, Supplemental Fig. 1). Skeletal-related events occurred in three patients: L5 compression fracture at 1 month in a patient with relapsed disease; L1 compression fracture at 5.5 months in a patient with relapsed disease; and rib fractures at 5.8 months in newly diagnosed disease for a 1-year SRE-free survival of 85.4% (95% CI 71.4–100%, Supplemental Fig. 2).

The most common toxicities of any grade on study were hypocalcemia (77.3%), infection (77.3%), fatigue (63.6%), and diarrhea (40.9%) (Table 2). The most common grade ≥3 adverse events included hypocalcemia (31.8%), neutropenia (31.8%), and infection (22.7%). Two patients developed suspected osteonecrosis of the jaw. One patient with relapsed disease developed grade 2 osteonecrosis of the jaw (ONJ) 10 months after starting denosumab. The second patient had newly diagnosed disease and developed grade 2 ONJ 11 days after starting denosumab (which may suggest pre-existing disease). No treatment-related deaths occurred.

**Table 2:** Adverse events.

Adverse event	Grade 1 N (%)	Grade 2 N (%)	Grade 3 N (%)	Grade 4 (%)	All N (%)	Grade 3-4 N (%)
Hypocalcemia	5 (22.7%)	5 (22.7%)	6 (27.3%)	1 (4.5%)	17 (77.3%)	7 (31.8%)
Infection	6 (27.3%)	6 (27.3%)	4 (18.2%)	1 (4.5%)	17 (77.3%)	5 (22.7%)
Fatigue	8 (36.4%)	6 (27.3%)	0 (0%)	0 (0%)	14 (63.6%)	0 (0%)
Diarrhea	8 (36.4%)	1 (4.5%)	0 (0%)	0 (0%)	9 (40.9%)	0 (0%)
Neutropenia	0 (0%)	1 (4.5%)	6 (27.3%)	1 (4.5%)	8 (36.4%)	7 (31.8%)
Pain	5 (22.7%)	1 (4.5%)	2 (9.1%)	0 (0%)	8 (36.4%)	2 (9.1%)
Nausea	5 (22.7%)	1 (4.5%)	1 (4.5%)	0 (0%)	7 (31.8%)	1 (4.5%)
Anemia	1 (4.5%)	2 (9.1%)	3 (13.6%)	0 (0%)	6 (27.3%)	3 (13.6%)
Cough	5 (22.7%)	0 (0%)	1 (4.5%)	0 (0%)	6 (27.3%)	1 (4.5%)
Fever	4 (18.2%)	1 (4.5%)	1 (4.5%)	0 (0%)	6 (27.3%)	1 (4.5%)
Dyspnea	4 (18.2%)	0 (0%)	1 (4.5%)	0 (0%)	5 (22.7%)	1 (4.5%)
Hyperkalemia	1 (4.5%)	1 (4.5%)	3 (13.6%)	0 (0%)	5 (22.7%)	3 (13.6%)
Hypertension	0 (0%)	2 (9.1%)	2 (9.1%)	0 (0%)	4 (18.2%)	2 (9.1%)
Lymphocyte count decreased	1 (4.5%)	0 (0%)	3 (13.6%)	0 (0%)	4 (18.2%)	3 (13.6%)
Hypophosphatemia	1 (4.5%)	0 (0%)	2 (9.1%)	0 (0%)	3 (13.6%)	2 (9.1%)

All adverse events occurring >20% and all grade 3–4 events occurring >5% are shown. There was one grade 4 infection due to COVID-19.

Hypocalcemia was managed with intravenous calcium in four patients (two of whom were on dialysis). Calcitriol was used in 7 patients (two of whom were on dialysis). Denosumab was held in 12 patients. Per protocol, there were no dose reductions for denosumab. Seven denosumab dose holds were for hypocalcemia; three were for osteonecrosis of the jaw or dental concerns; one was for pneumonia; and one was for other reasons. All seven patients with hypocalcemia-related dose holds resumed denosumab at the same schedule without dose reduction after a mean hold of 64 ± 14 days (range 56–93). One of these patients subsequently had an additional dose hold for hypocalcemia lasting 84 days.

Baseline serum CTX was significantly negatively correlated with the lowest serum calcium during treatment (Spearman *ρ* = −0.77, *p* = 0.0002, *n* = 18, Supplementary Fig. 3A). Patients on dialysis showed a trend toward higher likelihood of grade ≥2 hypocalcemia compared with those not on dialysis, 77.8% (7/9) versus 38.5% (5/13); OR 5.1, *p* = 0.099 by Fisher’s exact test (Supplementary Fig. 3B).

Denosumab carries a lower risk of nephrotoxicity than zoledronic acid, making it potentially well suited for patients with severe renal impairment, a common clinical scenario in MM. To our knowledge, this is the first prospective trial specifically evaluating denosumab in MM patients with creatinine clearance <30 mL/min. While there are several limitations in the study, such as small sample size, early study closure, and a mixed population of newly diagnosed and relapsed disease, several observations are noteworthy. Denosumab significantly lowered serum CTX by month 3 and had a 1-year SRE-free rate of 85.4%, similar to what was observed in patients with normal renal function [5, 6]. However, hypocalcemia was common and clinically significant, with grade ≥3 events occurring in 31.8%, and four patients required intravenous calcium. This rate of hypocalcemia is substantially higher than the 4% rate of grade ≥3 events seen in the randomized study of denosumab, which by design excluded patients with creatinine clearance <30 mL/min [5]. Notably, the hypocalcemia in our trial occurred despite mandated calcium and vitamin D supplementation and adequate 25-OH vitamin D levels prior to starting denosumab. Additional common adverse events observed in the trial, such as neutropenia and infection, are likely attributable to the concurrent MM therapy. The rates of these adverse events were similar between the denosumab and zoledronic arms of the randomized phase 3 trial in MM [5].

Indeed, renal insufficiency, particularly in the context of chronic kidney disease-mineral and bone disorder, is a risk factor for hypocalcemia with denosumab in general, as noted in the osteoporosis literature [7, 8]. To address this risk, the U.S. Food and Drug Administration added a warning in 2024 on using denosumab for treating osteoporosis in patients with chronic kidney disease [9]. In MM, the International Myeloma Working Group Bone Subcommittee recently presented findings on denosumab in 118 MM patients with renal insufficiency (median creatinine clearance 23.8 mL/min) and similarly found a high rate of grade ≥3 hypocalcemia, 22% [10]. The Subcommittee authors proposed a lower monthly dose of denosumab, 60 mg, to mitigate the risk of hypocalcemia.

For MM with renal insufficiency, close attention to risk of significant hypocalcemia is essential when using denosumab. Ongoing studies will be important to optimize the dosing and schedule of denosumab for this challenging patient population and to translate these findings into real-world practice [11].

## Supplementary information

Supplemental figures

## Data Availability

Data are available from the corresponding author on reasonable request.
