# Supplementary material for: Denosumab in multiple myeloma patients with severe renal impairment: a multicenter phase 2 study
Source: Blood Cancer J. 2026 Nov 2;16(1):195. doi: 10.1038/s41408-026-01656-y (PMC13728160; doi:10.1038/s41408-026-01656-y)
Supplement: Supplementary file 1 [file 41408_2026_1656_MOESM1_ESM.pdf]

Supplemental Figure 1

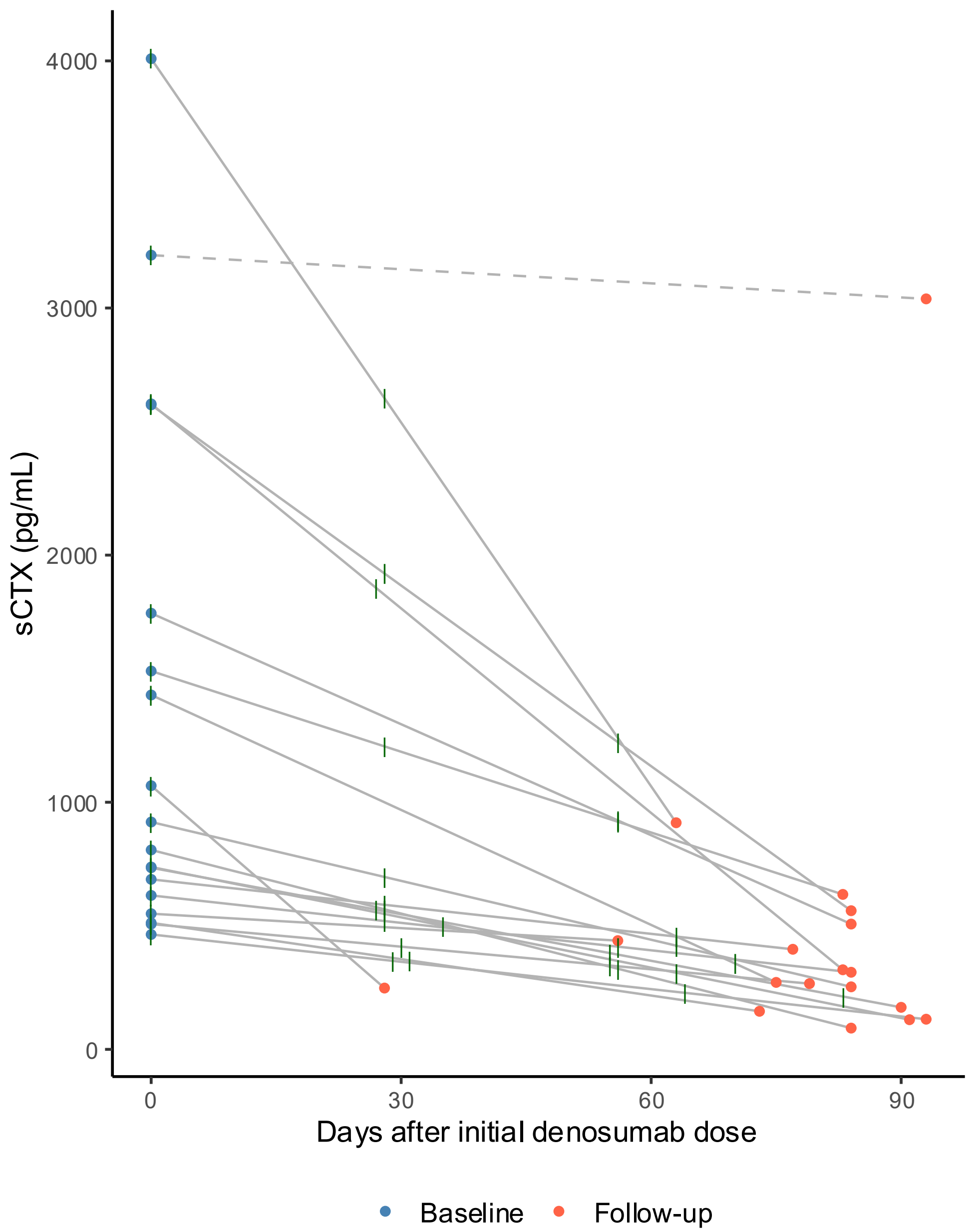

Change in serum CTX after denosumab administrations. Follow-up sCTX measurement was a median 83.5 days after a median of 3 (range 1-4) denosumab doses (n=18). Green tick marks indicate denosumab dosing. Dashed line indicates a patient with minimal serum CTX reduction who received only one dose of denosumab due to dose hold for hypocalcemia.

Supplemental Figure 2

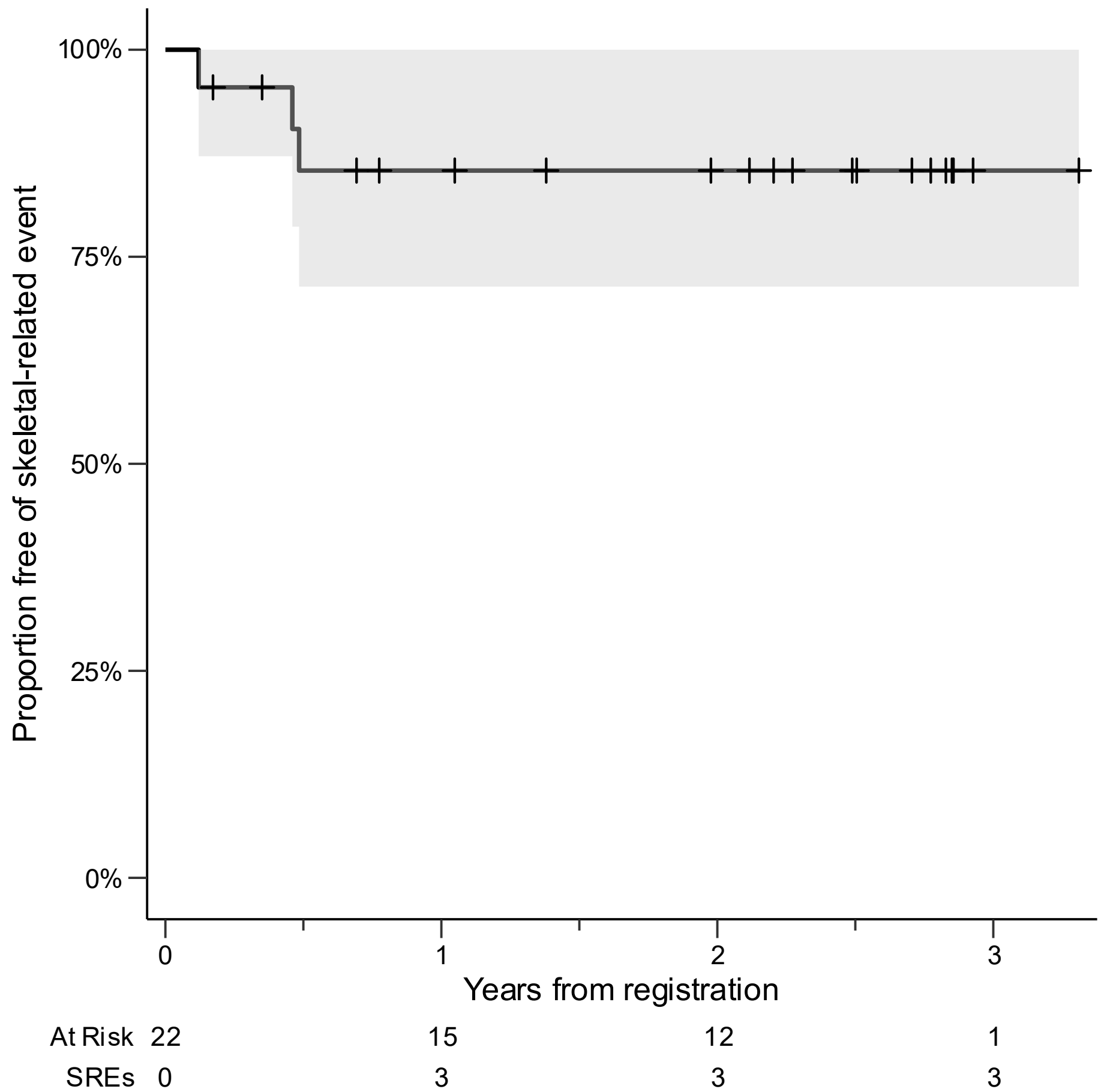

Kaplan-Meier estimate of time to first skeletal-related event.
1-year SRE-free survival 85.4% (95% CI 71.4-100%)

Supplemental Figure 3

A

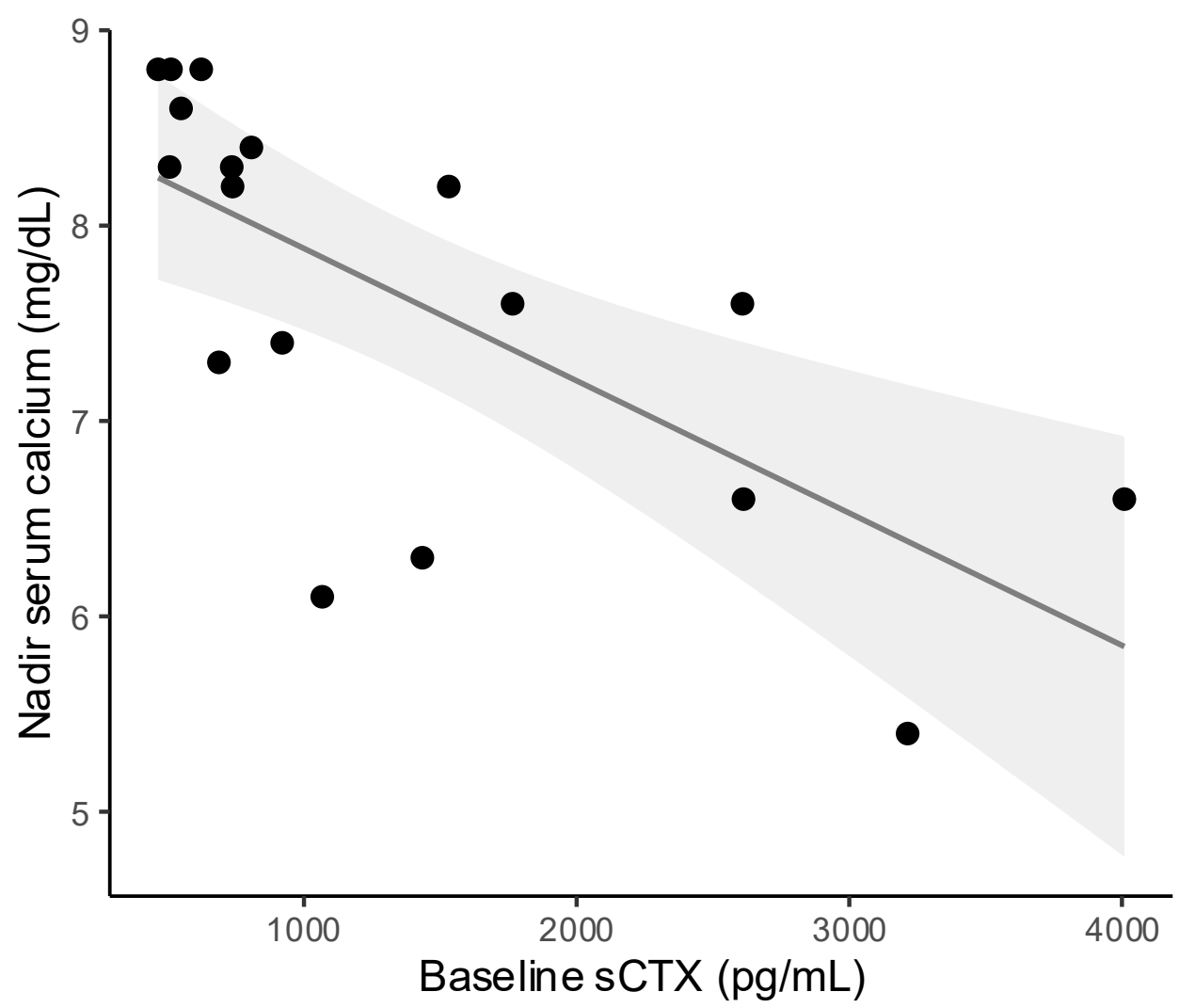

Nadir serum calcium versus baseline serum CTX. There was a significant negative correlation, Spearman ρ = −0.77, p = 0.0002, n = 18.

B

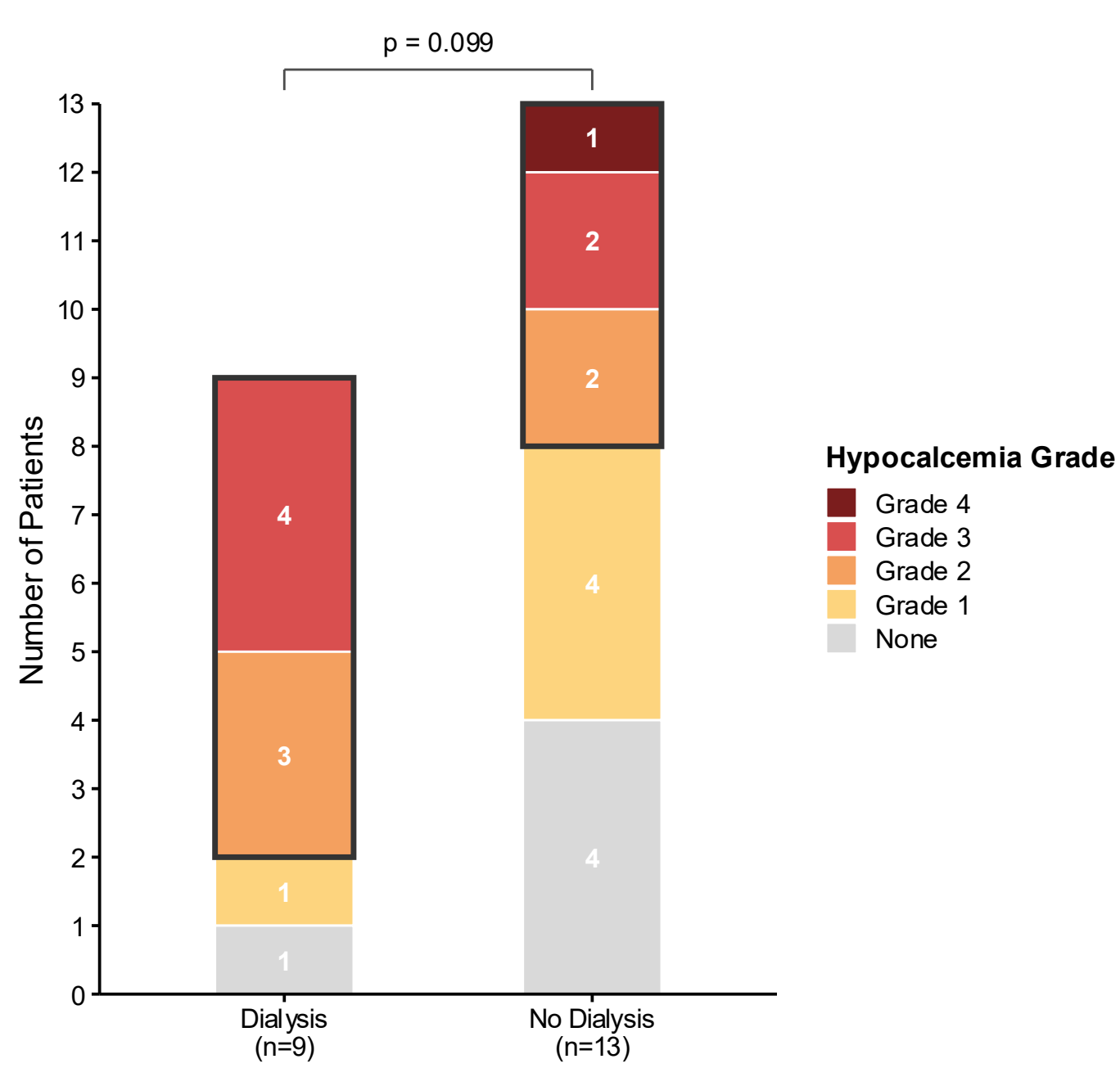

Frequency of hypocalcemia adverse events by dialysis status. Grade ≥2 hypocalcemia occurred in 77.8% (7/9) of patients on dialysis versus 38.5% (5/13) of patients not on dialysis, OR 5.1, p = 0.099 (Fisher's exact test).
